# Characterization and Significance of Monocytes in Acute Stanford Type B Aortic Dissection

**DOI:** 10.1155/2020/9670360

**Published:** 2020-05-15

**Authors:** Li Lu, Yuanhao Tong, Wenwen Wang, Yayi Hou, Huan Dou, Zhao Liu

**Affiliations:** ^1^Department of Vascular Surgery, Nanjing Drum Tower Hospital, The Affiliated Hospital of Nanjing University Medical School, Nanjing 210008, China; ^2^The State Key Laboratory of Pharmaceutical Biotechnology, Division of Immunology, Medical School, Nanjing University, Nanjing 210093, China; ^3^Department of Rheumatology and Immunology, Nanjing Drum Tower Hospital, The Affiliated Hospital of Nanjing University Medical School, Nanjing 210008, China; ^4^Jiangsu Key Laboratory of Molecular Medicine, Nanjing University, Nanjing 210093, China

## Abstract

Acute aortic dissection (AAD) is one of the most common fatal diseases noted in vascular surgery. Human monocytes circulate in dynamic equilibrium and display a considerable heterogeneity. However, the role of monocytes in AAD remains elusive. In our recent study, we firstly obtained blood samples from 22 patients with Stanford type B AAD and 44 age-, sex-, and comorbidity-matched control subjects. And the monocyte proportions were evaluated by flow cytometry. Results showed that the percentage of total CD14^+^ monocytes in the blood samples of Stanford AAD patients was increased significantly compared with that of normal volunteers (*P* < 0.0005), and the absolute numbers of CD14^bright^CD16^+^ and CD14^bright^CD16^−^ monocytes both increased significantly regardless of the percentage of PBMC or CD14^+^ cells, while CD14^dim^CD16^+^ monocytes displayed the opposite tendency. However, the percentage of CD14^+^ cells and its three subsets demonstrated no correlation with D-dimer (DD) and C-reactive protein (CRP). Then, blood mononuclear cell (PBMC) samples were collected by Ficoll density gradient centrifugation, followed with CD14^+^ magnetic bead sorting. After the purity of CD14^+^ cells was validated over 90%, AAD-related genes were concentrated in CD14^+^ monocytes. There were no significant differences observed with regard to the mRNA expression levels of *MMP1* (*P* = 0.0946), *MMP2* (*P* = 0.3941), *MMP9* (*P* = 0.2919), *IL-6* (*P* = 0.4223), and *IL-10* (*P* = 0.3375) of the CD14^+^ monocytes in Stanford type B AAD patients compared with those of normal volunteers. The expression levels of *IL-17* (*P* < 0.05) was higher in Stanford type B AAD patients, while the expression levels of TIMP1(P<0.05), TIMP2(P<0.01), *TGF-β1* (*P* < 0.01), *SMAD3* (*P* < 0.01), *ACTA2* (*P* < 0.001), and *ADAMTS-1* (*P* < 0.001) decreased. The data suggested that monocytes might play an important role in the development of Stanford type B AAD. Understanding of the production, differentiation, and function of monocyte subsets might dictate future therapeutic avenues for Stanford type B AAD treatment and can aid the identification of novel biomarkers or potential therapeutic targets for decreasing inflammation in AAD.

## 1. Introduction

Acute aortic dissection (AAD) is one of the most common emergencies of vascular surgery. A recent study demonstrated that the incidence of AAD was increased during the past decades [1, 2]. During the development of AAD, blood transfuses aorta through the ruptured aortic or blood vessels and separates the normal structure of the aorta, spreading into the media. This process results in the gradual expansion of the axial ends to form the true and false two-chamber state of the aorta, which is one of the most typical characteristics of AAD [3, 4]. In a recent study, AAD was divided into two types according to whether the ascending aorta was involved (type A) or not (type B) according to the Stanford system, which is a widely accepted classification system for AAD [5, 6]. Patients with Stanford type B AAD account for 25% to 40% of all aortic dissections and remain more likely to present with hypertension than those with Stanford type A AAD [7]. Furthermore, the majority of patients presenting with Stanford type A AAD are managed surgically (86% overall), while Stanford type B AAD is treated medically (63%), which makes our preclinical research of Stanford type B AAD more meaningful [8]. It has been also suggested that inflammation plays an important role in the development of AAD, which is receiving attention gradually [9, 10]. Several studies demonstrated that local inflammation was enhanced following the development of AAD, which was mainly reflected by the infiltration of large numbers of mononuclear macrophages, multinuclei leukocytes, and T/B lymphocytes [11, 12]. These inflammatory cells aggregate in the aorta and secrete inflammatory mediators to degrade the extracellular matrix, resulting in weakening of the aortic wall and reduced ability for gradual stress resistance [13]. In addition to the degradation of the extracellular matrix, local inflammation further causes ischemia, degeneration, and necrosis of aortic smooth muscle cells [14]. Flow dynamics (usually hypertension) eventually lead to rupture or dilation of the aortic intima, which in turn induces AAD [15]. Although several studies have been reported on the pathogenesis of AAD, it is well established that inflammation plays an important role in the development of this disease [16]. However, little is known with regard to the specific way by which inflammation participates in the development of Stanford type B AAD, especially the role of monocytes.

Monocytes are important cells of the innate immunity that are present in the circulation system. Based on the expression of the surface markers CD14 and CD16, a new nomenclature for dividing monocytes into three subgroups has been approved by the International Society of Immunology's Nomenclature Committee. This classification is the following: CD14^bright^CD16^−^ monocytes, CD14^bright^CD16^+^ monocytes, and CD14^dim^CD16^+^ monocytes [17]. The aforementioned cells circulate in dynamic equilibrium, and the kinetics underlying their production, differentiation, and disappearance are critical to understanding both homeostasis and inflammatory responses. Different monocyte subsets have different biological functions. CD14^bright^CD16^−^ monocytes are also known as classical monocytes, having superior phagocytosis activities. CD14^bright^CD16^+^ monocytes are also known as inflammatory monocytes, which are the inflammatory effector cells that stimulate the proliferation of T cells, the production of excessive reactive oxygen species (ROS), and the promotion of angiogenesis. CD14^dim^CD16^+^ monocytes are also known as nonclassical monocytes, used mainly for patrol of exogenous pathogens, as well as for antiviral defense [18, 19]. It was reported that monocytes recruited to areas of inflammation could differentiate into macrophages, which were involved in local aortic inflammatory responses in a mouse atherosclerosis model [20]. The inhibition of the differentiation of monocytes and the recruitment of mononuclear-macrophages to the aorta significantly improved the progression of atherosclerosis [21]. In addition, in abdominal aortic aneurysm (AAA), the accumulation of macrophages and the expression levels of the monocyte chemoattractant protein-1 (MCP-1) were both increased in the AAA wall [22, 23]. However, a limited number of studies have been performed with regard to the characteristics of monocytes in AAD. The specific mechanism of monocyte subsets with regard to the pathogenesis of AAD remains unclear. The purpose of the present study was to investigate the monocytic population and mediators on blood samples from patients with AAD.

## 2. Materials and Methods

### 2.1. Patient Eligibility Criteria

In the present study, 22 patients with AAD (Stanford type B) and 44 healthy volunteers were included. The acute Stanford type B aortic dissections are aortic dissections that arise when the entry tear is distal to the subclavian artery. The included criteria include the Acute Stanford type B aortic dissections which are one of the aortic dissections, which happened in 2 weeks and arise when the entry tear is distal to the subclavian artery. Acute Stanford B-type aortic dissection exclusion criteria are as follows: (1) Stanford B-type aortic dissection over 2 weeks, (2) aorta dissection involving the start of the left subclavian artery above the aorta, (3) the combination of immune diseases of Stanford type B aortic dissection, (4) Stanford type B aortic dissection patients with genetic diseases (such as Marfan syndrome), and (5) Stanford B-type aortic dissection after thoracic endovascular aortic repair (TEVAR) operation. In the parameters, gender, age, and history of hyperlipidemia did not exhibit a significant difference (*P* > 0.05) between the two groups, while significant differences were noted with regard to the variable history of diabetes (*P* < 0.05), smoking (*P* < 0.01), and hypertension (*P* < 0.001). All samples were collected from Nanjing Drum Tower Hospital, the Affiliated Hospital of Nanjing University Medical School. All volunteers provided written informed consent, and all studies were conducted according to the principles of the Declaration of Helsinki following approval by the relevant institutional review boards.

### 2.2. Flow Cytometry Analysis

All blood samples were transferred to the EDTA collecting tubes (Becton Dickinson). A total of 50 *μ*l blood was obtained and transferred into the corresponding flow tubes. The blood samples were treated with FC blocker (Miltenyi Biotec, Germany) at room temperature for 15 min and subsequently stained with Alexa Fluor 488-labeled anti-human CD14 (Miltenyi Biotec, Germany) and APC-labeled anti-human CD16 (Miltenyi Biotec, Germany). The aforementioned antibodies were incubated at 4°C for 30 min in the dark. Subsequently, the blood samples were treated with 1x FACS™ lysis solution for approximately 10 min, and FACS buffer was added. The samples were finally centrifuged at 300 g for 5 min at 4°C, rinsed again with FACS buffer, and finally resuspended in 150 *μ*l FACS buffer for subsequent testing by flow cytometry (BD Accuri™ C6, BD Biosciences, USA). All the antibodies were used according to the manufacturer's instructions. Classical monocyte cells were defined as CD14^bright^CD16^−^, whereas inflammatory monocyte cells were defined as CD14^bright^CD16^+^ and nonclassical monocyte cells as CD14^dim^CD16^+^.

### 2.3. Magnetic Bead Sorting

Blood samples were collected from 8 patients with AAD (Stanford type B) and 8 healthy volunteers. Total blood was isolated by a Ficoll density gradient centrifugation step in order to obtain peripheral blood mononuclear cells (PBMCs). The cell number was determined, and the cell suspension was centrifuged at 300 g for 10 min. The supernatant was aspirated completely, and the pellet was resuspended in 80 *μ*l buffer per 10^7^ of total cells. Subsequently, 20 *μ*l CD14 Microbeads (Miltenyi Biotec, Germany) was added per 10^7^ total cells, mixed, and incubated for 15 min at 2-8°C. The cells were washed by addition of 1-2 ml buffer per 10^7^ total cells and centrifuged at 300 g for 10 min. The supernatant was removed completely, and the cell pellet (10^7^ cells) was resuspended in 500 *μ*l buffer and further processed for magnetic separation. An appropriate MACS column and a MACS separator were selected according to the number of total cells and the number of CD14^+^ cells. LS columns (Miltenyi Biotec, Germany) were used for separation. Initially, the column was placed in the magnetic field of a suitable MACS separator (Miltenyi Biotec, Germany) and prepared by rinsing with 3 ml FACS buffer. The cell suspension was applied onto the column, and new FACS buffer was added when the column reservoir was empty. Subsequent washing steps were performed by adding 3 ml FACS buffer three times, and the column was removed from the separator following removal of the content in the column reservoir. The cell suspension was transferred to a suitable collection tube, and 5 ml FACS buffer was pipetted onto the column. The magnetically labeled cells were immediately removed by pushing the plunger into the column, and the collected cells were CD14^+^ cells. A minor fraction of the CD14^+^ cells was centrifuged at 300 g for 5 min at 4°C and resuspended in 100 *μ*l FACS buffer. The cells were stained with Alexa Fluor 488-labeled anti-human CD14 antibody (Alexa Fluor 488-CD14, Miltenyi Biotec, Germany) and incubated at 4°C for 30 min in the dark. The cells were further centrifuged at 300 g for 5 min at 4°C, resuspended in 150 *μ*l FACS buffer, and subsequently detected by flow cytometry (BD Accuri™ C6, BD Biosciences, USA). The remaining CD14^+^ cells were centrifuged at 300 g for 5 min, and following aspiration of the supernatant, they were subsequently tested.

### 2.4. Quantitative Polymerase Chain Reaction (RT-qPCR)

Total RNA from CD14^+^ cells were extracted by the TRIzol reagent (Invitrogen, USA) and reverse-transcribed by a reverse transcription kit (HiScript® II Q RT SuperMix for qPCR, Vazyme Biotech, Nanjing, China). Subsequently, the cDNAs were used as templates for RT-qPCR analysis performed on the BIOER Line Gene 9640 detection system (Hangzhou, China). The Ct value and the relative expression levels of each gene were calculated according to the 2^-*ΔΔ*Ct^ formula. The relative amount of the target gene and that of the reference gene *GAPDH* were obtained. All the reactions were repeated three times. All RNA samples exhibited a 260/280 ratio of ≈2.0. The primer sequences used are shown in Table 1.

### 2.5. Statistical Analysis

The data were expressed as the mean ± SEM. Statistical analyses were performed by GraphPad Prism 5 (San Diego, CA, USA). Multigroup comparisons were analyzed by Student's *t*-test or the one-way ANOVA test. A *P* value less than 0.05 (*P* < 0.05) was considered for significant differences. The experiments were repeated at least three times.

## 3. Results

### 3.1. Clinical Characteristics of the Patients with AAA

The D-dimer values and the CRP values in Stanford type B AAD patients were higher than those noted in healthy control subjects (*P* < 0.001), while there was significant difference in history of diabetes (*P* < 0.05), smoking (*P* < 0.01), and hypertension (*P* < 0.001). In addition, apparent differences were noted in the percentage of neutrophils (*P* < 0.01), lymphocytes (*P* < 0.001), WBC count, and monocytes (*P* < 0.001) between these two groups (Table 2).

### 3.2. The Behaviors of Monocyte Subsets in Stanford Type B AAD

**Figure 1:**
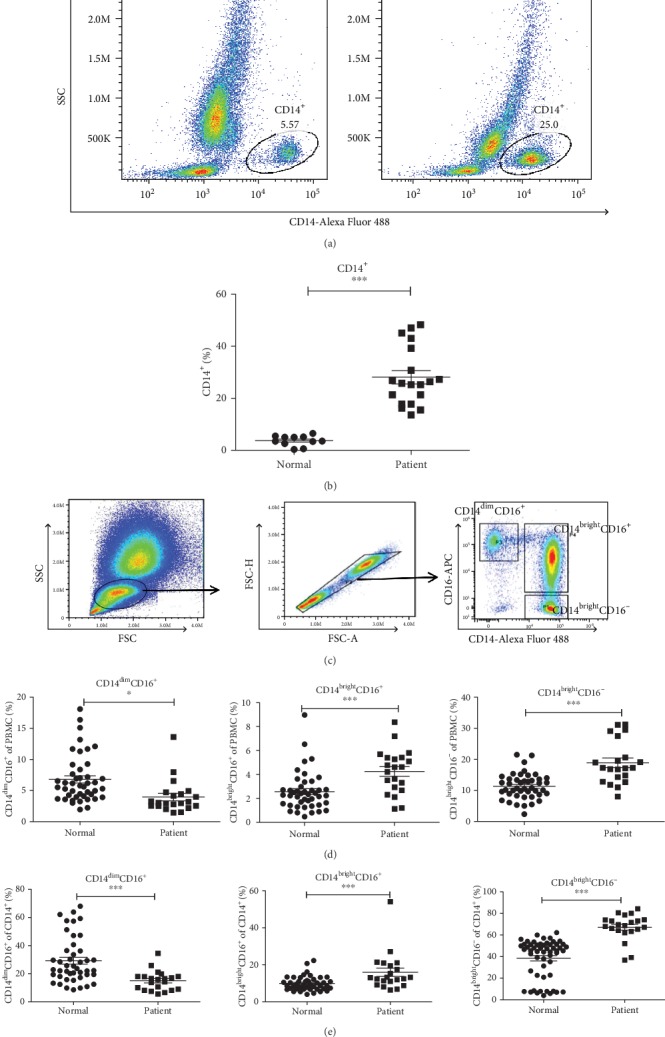
(a) Flow cytometry analysis of CD14^+^ monocytes in blood of normal control and Stanford type B AAD patients; (b) the statistical graph of (a); (c) gating strategy of monocyte subsets via flow cytometry: CD14^bright^CD16^−^ (classic monocytes), CD14^bright^CD16^+^ (intermediate), and CD14^dim^CD16^+^ (nonclassical); (d) the statistical graph in the percentage of three monocyte subsets in PBMC; (e) the statistical graph in the percentage of three monocyte subsets in CD14^+^ monocytes; data were shown as the mean ± SEM (standard error of the mean). Stanford type B AAD patient: *n* = 22; normal control *n* = 44. The data were means ± SEM. ^∗^*P* < 0.05, ^∗∗^*P* < 0.01, and ^∗∗∗^*P* < 0.005 vs. normal group.

Currently, numerous studies have demonstrated that aortic inflammation is inseparable for the development of aortic disease and that monocytes play an important role in inflammation. Following the induction of inflammation in local tissues, monocytes are recruited to the tissues and differentiate into macrophages, which secrete inflammatory mediators and participate in local inflammatory reactions [24–26]. In order to demonstrate the population of monocytes in Stanford type B AAD patients, we performed flow cytometry analysis with human blood samples. The results indicated that the percentage of CD14^+^ cells in AAD patients was significantly higher than that of normal volunteers (Figures 1(a) and 1(b)). To further confirm the biological features of the three monocyte subsets, we analyzed their percentages in PBMCs. The gating strategy is shown in Figure 1(c) [27]. The results indicated that CD14^dim^CD16^+^ monocytes exhibited a significant decrease in the proportion of PBMCs (*P* < 0.05), while the percentages of the two other monocyte subsets (CD14^bright^CD16^−^, CD14^bright^CD16^+^) were increased significantly (*P* < 0.001) (Figure 1(d)). Due to the significant increase caused in the percentage of CD14^+^ cells in PBMC (*P* < 0.001), we analyzed the percentages of the three monocyte subsets in CD14^+^ cells.

### 3.3. Correlation Analysis between Monocyte and D-Dimer and C-Reactive Protein

**Figure 2:**
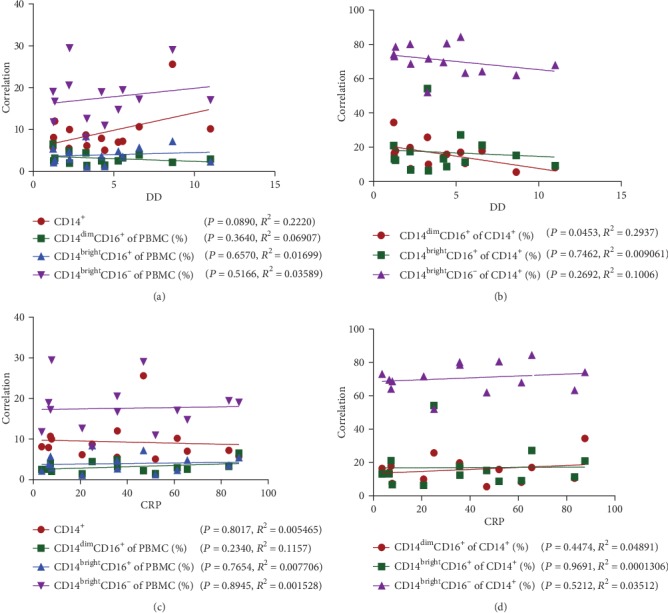
(a) Correlation analysis between the serum levels of DD and the percentage of monocyte subsets (CD14^dim^CD16^+^, CD14^bright^CD16^+^, and CD14^bright^CD16^−^) in PBMC. *P* > 0.05. (b) Correlation analysis between the serum levels of DD and the percentage of monocyte subsets (CD14^dim^CD16^+^, CD14^bright^CD16^+^, and CD14^bright^CD16^−^) in CD14^+^ cells. *P* > 0.05. (c) Correlation analysis between the serum levels of CRP and the percentage of monocytes subsets (CD14^dim^CD16^+^, CD14^bright^CD16^+^, CD14^bright^CD16^−^) in PBMC. *P* > 0.05. (d) Correlation analysis between the serum levels of CRP and the percentage of monocyte subsets (CD14^dim^CD16^+^, CD14^bright^CD16^+^, and CD14^bright^CD16^−^) in CD14^+^ cells. *P* > 0.05; *n* = 14. The data were means ± SEM. ^∗^*P* < 0.05, ^∗∗^*P* < 0.01, and ^∗∗∗^*P* < 0.005 vs. normal group.

DD and CRP were viewed as diagnostic and prognostic tools for AAD. D-dimer is a fibrin degradation product, generated following fibrinolysis of a thrombus [28–30]. C-reactive protein (CRP) is an acute phase reactant, which is a sensitive and a nonspecific inflammatory marker [30–33]. The serum levels of DD and CRP are usually used to assess the overall severity of acute diseases or to predict adverse events [34, 35]. The correlation analysis indicated no significant correlation between DD and the percentages of monocyte subsets in PBMCs (all *R*^2^ < 0.5 and *P* > 0.05) (Figure 2(a)). Similar results were observed with regard to the correlation between DD and the percentages of monocyte subsets in CD14^+^ cells (all *R*^2^ < 0.5 and *P* > 0.05) (Figure 2(b)), while a weaker correlation between CRP and the percentages of monocyte subsets was noted in the present study (all *R*^2^ < 0.5 and *P* > 0.05) (Figures 2(c) and 2(d)).

### 3.4. CD14^+^ Monocyte Gene Detection

**Figure 3:**
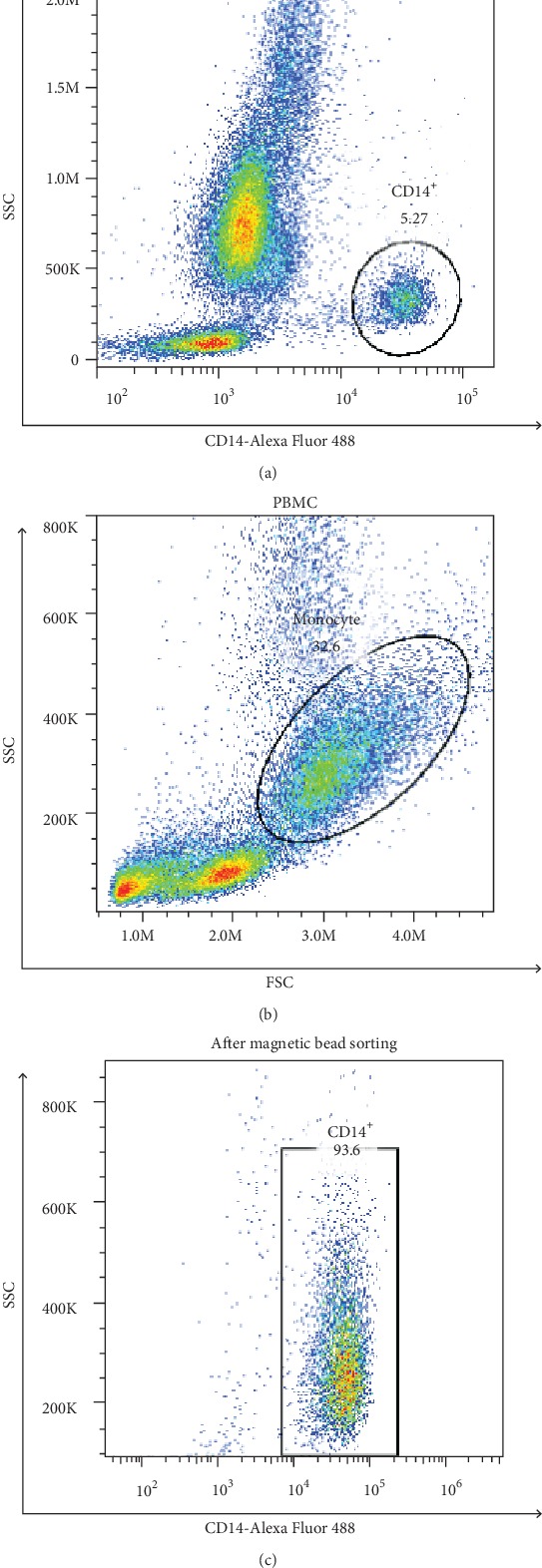
(a) Flow cytometry analysis of CD14^+^ cells in blood; (b) flow cytometry analysis of CD14^+^ cells in PBMC; (c) flow cytometry analysis of CD14^+^ cells after magnetic bead sorting.

**Figure 4:**
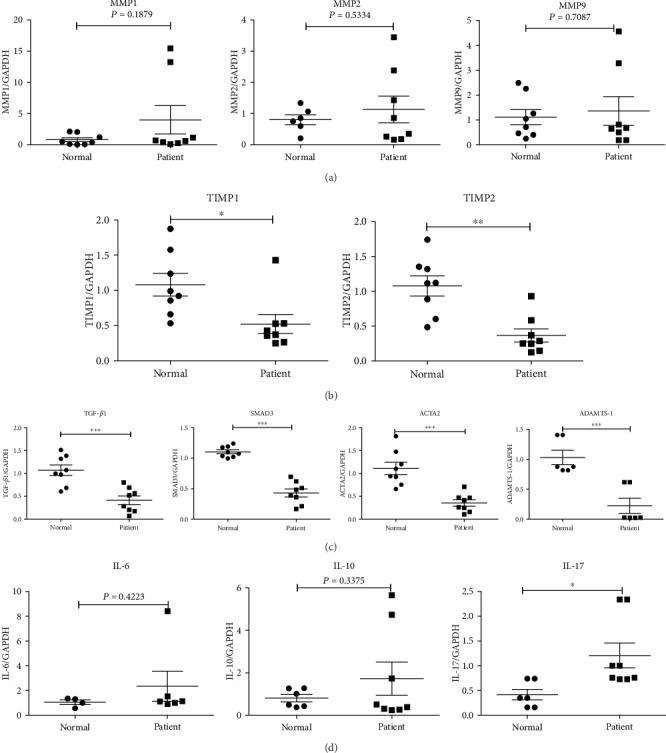
(a) The mRNA expression levels of MMP1, MMP2, and MMP9; (b) the mRNA expression levels of TIMP1, TIMP2; (c) the mRNA expression levels of TGF-*β*1, SMAD3, ACTA2, and ADAMTS-1; (d) the mRNA expression levels of IL-6, IL-10, and IL-17; Stanford type B AAD patient: *n* = 8; normal control: *n* = 8. The data were means ± SEM. ^∗^*P* < 0.05, ^∗∗^*P* < 0.01, and ^∗∗∗^*P* < 0.005 vs. normal group.

To further explore the characteristics of monocytes in Stanford type B AAD patients, we attempted to obtain human CD14^+^ monocytes by magnetic bead sorting. The percentage of CD14^+^ monocytes was approximately 5% of the total cells (Figure 3(a)). Following gradient density centrifugation, the percentage of CD14^+^ monocytes in PBMCs increased to approximately 30% (Figure 3(b)). Finally, the purity of CD14^+^ monocytes was higher than 90% following magnetic bead sorting (Figure 3(c)). On this basis, we detected several genes by RT-qPCR, which were important for the development of AAD in CD14^+^ monocytes. The results indicated that the expression levels of the matrix metalloproteinase family genes (*MMP1*, *MMP2*, and *MMP9*) were not significantly different between Stanford type B AAD patients and normal volunteers (Figure 4(a)). The expression levels of the tissue inhibitor matrix metalloproteinase genes (*TIMP1* and *TIMP2*) were decreased (*P* < 0.05) (Figure 4(b)). Similarly, the levels of transforming growth factor-*β*1 (*TGF-β1*), SMAD3, alpha-actin (*ACTA2*), and a disinterring and metalloproteinase with thrombospondin motifs 1 (*ADAMTS-1*) were decreased (Figure 4(c)). The mRNA expression levels of *IL-6* and *IL-10* indicated no significant difference between Stanford type B AAD patients and normal volunteers, while *IL-17* levels were apparently increased (Figure 4(d)).

## 4. Discussion

Acute aortic dissection (AAD) is the most serious clinical emergency in aortic disease. It exhibits an acute onset, rapid development, morbidity complications, a high mortality rate, and an increased misdiagnosis rate [4, 5, 36]. Additional studies have contributed significantly to the prevention, diagnosis, and treatment of AAD diseases. In the present study, we demonstrated that the percentages of total monocytes and the subsets in the blood samples of Stanford type B AAD patients were markedly altered, although no apparent correlation with DD and CRP was noted. However, several genes associated with AAD (*TIMP1*, *TIMP2*, *TGF-β1*, *SMAD3*, *ACTA2*, *ADAMTS-1*, and *IL-17*) demonstrated significant changes in their levels in Stanford type B AAD patients. The investigation of the number of CD14^+^ monocytes is required to further understand the characteristics of monocytes in AAD.

It is well known that the aorta is composed of a large number of extracellular matrix components for the maintenance of the arterial blood flow and the blood pressure. Following degradation of these structures, the arterial wall is dilated and ruptures [3, 37]. Recent studies have demonstrated that multiple inflammatory cells are involved in the remodeling of aortic vascular tissues, such as activated T and B cells and mononuclear-macrophages [38–40], indicating that inflammation participates in the development of AAD by regulating the number, location, and functional balance of the inflammatory cells. As one of the most important inflammatory cells, monocytes can participate in the vascular remodeling process via different mechanisms of action. However, a limited number of studies have explored these pathways. Previous studies have reported that the percentage of monocytes in blood of Stanford A AAD patients was increased [41], while there were no related reports in patients with Stanford type B AAD. The results of the present study indicated by flow cytometry that the percentage of CD14^+^ cells in the blood of Stanford type B AAD patients was higher than that of normal volunteers. The findings indicated that monocytes were multiplied and recruited to areas of inflammation where they differentiated and acted as effector cells to respond to various biological changes [42].

From 2010, a new nomenclature for classifying monocytes into three subgroups has been approved by the International Society of Immunology's Nomenclature Committee. This classification is based on the expression levels of the surface markers CD14 and CD16 and is the following: CD14^bright^CD16^−^, CD14^bright^CD16^+^, and CD14^dim^CD16^+^ [16]. CD14^bright^CD16^−^ monocytes comprise 80–90% of the monocyte pool with the remaining 10-20% being shared by CD14^bright^CD16^+^ and CD14^dim^CD16^+^ monocytes [43]. The features of these three types of monocytes were examined in Stanford type B AAD patients. The monocytes were gated according to their CD14 and CD16 expression, and the results demonstrated that the percentages of CD14^dim^CD16^+^ monocytes in PBMC and in CD14^+^ cells were significantly decreased in AAD patients, while the percentages of the other two subsets (CD14^bright^CD16^−^ and CD14^bright^CD16^+^) were markedly increased. Similar results were observed in other types of diseases. For example, the number of CD14^bright^CD16^+^ monocytes in patients with coronary heart disease was higher and exhibited a positive correlation with atherogenic plaque formation [44]. A higher increase of CD14^bright^CD16^+^ monocytes and lower levels of CD14^bright^CD16^−^ monocytes in patients with acute takotsubo cardiomyopathy has been previously shown [45]. Tsujioka et al. demonstrated that the peak levels of CD14^bright^CD16^−^ monocytes exhibited a negative association with the recovery of left ventricular function following acute myocardial infarction [46]. CD14^bright^CD16^−^ and CD14^bright^CD16^+^ monocytes are inflammatory effectors recruited in inflammatory sites in response to inflammatory stimuli. It has been shown that the CD14^bright^CD16^−^ monocytes mature via a continuum to CD14^bright^CD16^+^ monocytes and subsequently to CD14^dim^CD16^+^ monocytes [47]. We proposed that the decrease of CD14^dim^CD16^+^ monocytes might be due to the reduced maturation of CD14^bright^CD16^−^ monocytes [18]. Furthermore, we considered that the infiltration of the macrophages detected in the aorta might result from the migration of circulating monocytes, rather than the localization of the resident aorta macrophages. They are involved in the remodeling of aortic blood vessels. Therefore, the increase in the percentages of these two monocytes was cognitively compatible. It was hypothesized that the reduction in the number of noncanonical monocytes was mediated by the decreased maturation of classical monocytes. The maturation pathway of monocytes involves the maturation of CD14^bright^CD16^−^ monocytes to CD14^bright^CD16^+^ [17, 27]. In summary, the results indicated that these three monocyte subsets might play different roles in the development of Stanford type B AAD.

D-dimer (DD) is a specific protein fiber degradation product that is formed by plasmin hydrolysis. DD is released in large quantities following thrombosis, resulting in elevated levels of this biomarker in the serum [48, 49]. Therefore, the serum levels of DD are considered an optimal diagnostic tool for deep vein thrombosis, pulmonary embolism, and AAD [50–52]. This assessment was well recognized in the diagnosis of acute aortic syndrome (including AAD) [1, 52–54]. In a previous study conducted in 2006, D-dimer testing (DT) was performed in 113 consecutive AAD patients within 24 h of symptom onset in the Osaka Mishima Emergency and Critical Care Center [55]. The results indicated that 104 (92%) AAD patients were positive for DT [55]. In the same year, the University Hospital of Strasbourg in France performed DT in 94 consecutive patients admitted to their institution with confirmed AAD, and the results indicated that 93 patients (99%) with AAD exhibited elevated DD (>400 ng/ml) [55]. It has been shown that the serum levels of DD are positively correlated with AAD. However, the present study indicated no significant correlation between DD and the percentages of monocyte subsets. It is possible that the two are independent factors affecting the disease process. In addition, it may be related to the sampling time point. A previous study demonstrated that the positive rate of DT detection was considerably low in patients aged less than 70 years and in patients exhibiting a 120 min time interval from symptom onset to admission [56]. The mean age of the patients of the present study was lower than 70 years, and the time interval from symptom onset to blood sample acquisition and detection was approximately 120 h, suggesting that this may be an important cause of negative results. In addition, we hypothesized that the time point required for the change in the monocyte number in Stanford type B AAD was different from that noted for the increase in the DD levels in other inflammatory diseases. The number of monocytes was increased in response to the changes in the microenvironment occurring during the disease progression. However, the large quantities of DD were released following induction of thrombosis. In addition to DD, C-reactive protein (CRP) is usually used to analyze the prognosis of AAD patients. The results indicated no significant correlation between CRP levels and monocyte subsets. CRP is a nonspecific acute inflammatory response protein produced by hepatocytes and is usually used to assess the overall severity of an acute disease or to predict adverse events [57]. However, it cannot be used as a clinical indicator for the prediction of AAD [57]. Furthermore, the time point required for the monocyte changes to take place may also differ from that noted for the CRP increase in the Stanford type B AAD patients, which remained to be confirmed by further research.

As we can see, diabetes (*P* < 0.05), smoking history (*P* < 0.01), and hypertension (*P* < 0.001) in Stanford type B AAD patients were higher than those noted in healthy control subjects. These might be related to the abnormal expression of monocytes and the occurrence of Stanford type B AAD. Actually, studies have been reported that the occurrence of male abdominal aortic aneurysms is closely related to the lifestyle-related factors, including cigarette smoking, obesity (body mass index (BMI) and waist circumference (WC)), and history of comorbidities (diabetes mellitus, hypercholesterolemia, and hypertension) [58]. What is the relationship between these lifestyle-related factors and the abnormal expression of monocytes? Studies have shown that the number of monocytes was increased in the blood of smokers [59]. Similarly, an increase in circulating monocyte counts has been found in patients with diabetes [60]. It has been reported that humans with hypertension have increasing intermediate and nonclassical monocytes, and increased endothelial stretch enhanced monocyte conversion to CD14^bright^CD16^+^ monocytes [61]. Thus, we can speculate that the above lifestyle-related factors may result in high expression of monocytes and induce Stanford type B AAD, which remains to be confirmed further.

To determine the biological changes caused on monocytes other than their percentages and phenotype, we obtained CD14^+^ monocytes by magnetic bead sorting and analyzed the expression levels of several genes of CD14^+^ cells. It has been shown that the expression levels of several genes are associated with the induction of aortic diseases induced by ANG II, such as *TGF-β* [62, 63], *SMAD3* [64–66], *MMP1* [66–69], *MMP2* [70–72], *MMP9* [72, 73], and *TIMP1/TIMP2* [74, 75]. Previous reports have clearly demonstrated that *MMP1*, *MMP2*, and *MMP9* are activated in the aortic disease [66–72]. However, the present study demonstrated that the expression levels of *MMP1*, *MMP2*, and *MMP9* indicated no significant differences between CD14^+^ cells in Stanford type B AAD patients and normal volunteers, while *TIMP1* and *TIMP2* (inhibitor of MMPs) levels were decreased [73–75]. It has been reported that the balance between MMPs/TIMPs regulates ECM conversion and remodeling, and an imbalance in this proportion may result in an abnormal amount of ECM degradation, leading to the development of severe vascular disease [76]. We speculated that monocytes may exhibit altered levels of TIMPs, but not of MMPs during the development of Stanford type B AAD.

Approximately 10 years ago, a hypothesis was proposed suggesting that aortic lesions were caused by excess TGF-*β*1 production in the aortic medium [77]. Subsequent studies confirmed this hypothesis and revealed that the downstream pathway of TGF-*β*1 was mediated by SMAD3. This suggested that excessive TGF-*β*/SMAD3 signaling stimulated smooth muscle cell uncontrolled and excessive proliferation, resulting in arterial lumen changes and the trigger of a series of aortic diseases [78, 79]. However, the present results indicated that the mRNA expression levels of *TGF-β1* and *SMAD3* were significantly decreased in Stanford type B AAD compared with those of normal volunteers, which was inconsistent with previous reports. Considering that the detection of CD14^+^ monocytes was obtained from the patient blood, these two genes involved in CD14^+^ monocyte function may play additional undiscovered roles, which require further investigation.

Alpha-actin (*ACTA2*) is the most common mutated gene in aortic disease, mainly affecting vascular smooth muscle cell (SMC) function. The major function of these cells is to contract in response to the stretch, a process that depends on the cyclic interaction between thin filaments, which are encoded by ACTA2 [80, 81]. The heterozygous mutations in ACTA2 lead to an inherited predisposition for thoracic aortic aneurysms and dissections (TAAD) [80–82]. The recent results of the present study indicated that the expression levels of ACTA2 in CD14^+^ monocyte were lower in Stanford type B AAD patients, suggesting that *ACTA2* may be mutated in CD14^+^ monocytes.

The recently discovered extracellular metalloproteinase named ADAMTS-1 is disinterring with a thrombospondin motif and metalloproteinase [83]. It has been reported that ADAMTS-1 inhibits ECM remodeling and participates in vascular disease by inhibiting cell proliferation [84–86]. Its mechanism of action is mediated by the combination of the vascular endothelial growth factor and the fibroblast growth factor [84–86]. Previous studies have demonstrated that the expression levels of ADAMTS-1 are increased significantly in aortic tissues of AAD patients [87, 88]. ADAMTS-1 was also introduced as a major mediator of vascular homeostasis, and ADAMTS-1^−/−^ mice were more susceptible to the AAA phenotype [89]. The results of the present study indicated that the expression levels of ADAMTS-1 in CD14^+^ cells were lower in Stanford type B AAD patients compared with those of the normal volunteers.

Interleukin-6 (IL-6) is an important stimulator of atherosclerotic lesions, which can aggravate atherosclerosis [90, 91]. However, the results of the current study indicated that the mRNA levels of IL-6 in Stanford type B AAD patient CD14^+^ cells exhibited no significant difference compared with those of the normal volunteers. In addition to IL-6, IL-10 also played an important role in promoting the development of aortic aneurysm. It has been reported that high levels of IL-10 are detected in the serum of patients with aneurysms and that IL-10^−/−^ counteracts Ang II-induced vascular dysfunction in APOE^−/−^ mice [92]. The results of the present study indicated that in CD14^+^ monocytes, the mRNA expression levels of IL-10 demonstrated no significant difference between Stanford type B AAD patients and normal volunteers, while interleukin-17 (IL-17) exhibited a significant increase. It has been reported that IL-17 is involved in various autoimmune diseases, including multiple sclerosis, rheumatoid arthritis, and systemic lupus erythematosus [[93–95], and previous studies have also indicated that high expression of IL-17 induces vascular inflammation, endothelial dysfunction, arterial hypertension, hypertension, and aortic aneurysm [96, 97]. In addition, IL-17 affects the basic function of the mononuclear/macrophage lineage and participates in the development of advanced atherosclerosis, promoting the increase of monocyte adhesion and the recruitment of circulating monocytes [98, 99]. We have demonstrated that the expression levels of IL-17 in CD14^+^ monocytes were increased in Stanford type B AAD patients and that this effect may be inextricably linked to the changes of the monocyte biological characteristics. This requires further investigation in further study.

Although the present study contains several limitations, the results reported that the numbers and phenotypes of monocytes were significantly altered in the blood of Stanford type B AAD patients, suggesting that monocytes may play an important role in promoting the development of Stanford type B AAD. The specific mechanism of this process remains undiscovered. The mRNA expression levels of CD14^+^ monocytes can aid in identification and prognosis of the disease. Understanding the characteristics of monocyte subset generation, differentiation, and function can dictate the development of future therapeutic avenues for Stanford type B AAD.

## Figures and Tables

**Table 1:** Primer sequences for quantitative real-time polymerase chain reaction.

Gene name	Forward primer	Reverse primer
MMP9	GGACGATGCCTGCAACGT	CAAATACAGCTGGTTCCCAATCT
MMP1	CATGAAAGGTGGACCAACAATTT	CCAAGAGAATGGCCGAGTTC
MMP2	TACACCAAGAACTTCCGTCTGT	AATGTCAGGAGAGGCCCCATA
TGF-*β*1	CTAATGGTGGAAACCCACAACG	TATCGCCAGGAATTGTTGCTG
SMAD3	ACCATCCCCAGGTCCCTGGATGGCC	AACTCGGCCGGGATCTCTGTGTGGCGT
ACTA2	TCAATGTCCCAGCCATGTAT	CAGCACGATGCCAGTTGT
TIMP1	CTTCTGCAATTCCGACCTCGT	ACGCTGGTATAAGGTGGTCTG
TIMP2	AAGCGGTCAGTGAGAAGGAAG	GGGGCCGTGTAGATAAACTCTAT
IL-6	ACTCACCTCTTCAGAACGAATTG	CCATCTTTGGAAGGTTCAGGTTG
IL-10	TCAAGGCGCATGTGAACTCC	GATGTCAAACTCACTCATGGCT
IL-17	TCCCACGAAATCCAGGATGC	GGATGTTCAGGTTGACCATCAC
ADAMTS-1	CAGAGCACTATGACACAGCAA	AGCCATCCCAAGAGTATCACA
GAPDH	AGAAGGCTGGGGCTCATTTG	AGGGGCCATCCACAGTCTTC

**Table 2:** Clinical data of patients.

Characteristics	Healthy control (*n* = 44)	AAD patient (*n* = 22)	*χ* ^2^ or *t*	*P* value
Mean age	52.89 ± 15.24	55.33 ± 13.32	0.744	0.459
Gender (M/F)	25/19	15/7	2.652	0.103
Smoking history	10 (22.73%)	12 (54.55%)	6.682	0.009^∗∗^
Hypertension (%)	9 (20.45%)	14 (63.64%)	12.05	<0.001^∗∗∗^
Hyperlipidemia (%)	12 (27.27%)	9 (40.91%)	1.257	0.262
Diabetes (%)	2 (4.54%)	6 (27.27%)	4.991	0.026^∗^
DD (mg/l)	0.26 ± 0.15	3.73 ± 2.54	8.875	<0.001^∗∗∗^
CRP (mg/l)	2.18 ± 1.27	30.57 ± 24.76	7.478	<0.001^∗∗∗^
WBC (10^9^/l)	6.38 ± 1.42	10.48 ± 2.02	9.368	<0.001^∗∗∗^
NE (%)	59.92 ± 6.81	67.21 ± 10.63	3.319	0.002^∗∗^
LY (%)	33.12 ± 6.93	24.25 ± 6.83	4.853	<0.001^∗∗∗^
MO (%)	5.64 ± 1.12	9.21 ± 2.32	8.276	<0.001^∗∗∗^

DD: D-dimer; CRP: C-reactive protein; NE (%): neutrophil percentage; LY (%): lymphocyte percentage; MO (%): monocyte percentage. Values were expressed as the mean ± SD or as indicated. The data of D-dimer was detected by an automatic blood coagulation analyzer (CA7000, Sysmex, Inc., Japan), and the data of CRP was analyzed by an automatic biochemical analyzer (C8000, Abbott, Inc., USA). The data were means ± SEM. ^∗^*P* < 0.05, ^∗∗^*P* < 0.01, and ^∗∗∗^*P* < 0.005 vs. healthy control.

## Data Availability

The data used to support the findings of this study are included within the article.
